# The complete chloroplast genome of *Symphoricarpos orbiculatus* (Caprifoliaceae), an important ornamental plant

**DOI:** 10.1080/23802359.2019.1668310

**Published:** 2019-09-24

**Authors:** Xue-Li Shen, Yi-Xuan Zhu, Yuan-Mi Wu, Ling Tong, Xian-Yun Mu

**Affiliations:** Laboratory of Systematic Evolution and Biogeography of Woody Plants, College of Nature Conservation, Beijing Forestry University, Beijing, P. R. China

**Keywords:** Symphoricarpos orbiculatus, complete chloroplast genome, illumina sequencing

## Abstract

*Symphoricarpos orbiculatus* is an important landscape and ornamental plant. In this study, we report the complete chloroplast genome sequences of *S. orbiculatus*. The complete chloroplast genome of *S. orbiculatus* was 156,044 bp in length. The genome has a typical quadripartite structure, including a large single-copy (LSC) region of 88,756 bp, a small single-copy (SSC) region of 19,130 bp, and two inverted repeat (IR) regions of 24,079 bp each. Overall, the GC content was 38.4%. In the genome, it was identified to comprise130 genes, including 85 protein-coding genes, 37 tRNA genes, and 8 rRNA genes. This study provides valuable information for molecular phylogenetic study of Caprifoliaceae and is significant for variety development of *Symphoricarpos*.

*Symphoricarpos* is native to North America, Mexico and China. This genus, with many garden hybrids, has been more and more widely used in garden landscape for its ornamental value and high adaptability. The inherited characteristic study for *Symphoricarpos* is significant to the development of new varieties of this genus. Furthermore, it will provide valuable information for molecular phylogenetic study of Caprifoliaceae. Here, we assembled the complete chloroplast genome of *Symphoricarpos orbiculatus* and the annotated chloroplast genome sequence has been deposited in GenBank with Accession Number MK970589.

The fresh leaves of *S. orbiculatus* were collected from Shanghai Chen Shan Botanical Garden (N31°04′48.10″, E121°11′5.76″). Voucher specimen (collector and collection number: *Xian-Yun Mu 3878*) is deposited in the herbarium of Beijing Forestry University. Genomic DNA extraction and next-generation sequencing were performed with an Illumina Hiseq platform. In total, 14.4 Gb of 150-bp clean reads were generated and used for chloroplast genome assembly through Geneious 11.1.4 software (Kearse et al. 2012) with *Lonicera japonica* (Kang et al. 2018) as a reference sequence. The assembled chloroplast genome was then annotated using the Plann (Huang and Cronk 2015). Eventually, annotations were verified in Geneious 11.1.4 software.

The complete chloroplast genome of *S. orbiculatus* was 156,044 bp in length. The genome has a typical quadripartite structure, including a large single-copy (LSC) region of 88,756 bp, a small single-copy (SSC) region of 19,130 bp, and two inverted repeat (IR) regions of 24,079 bp each. Overall, the GC content was 38.4%. In the *S. orbiculatus* chloroplast genome, it was identified to comprise130 genes, including 85 protein-coding genes, 37 tRNA genes, and 8 rRNA genes.

*S. orbiculatus* and other 6 species from Caprifoliaceae, with 2 species from Adoxaceae as outgroup species, were used for phylogenetic analysis (Figure 1). A total of nine complete chloroplast genomes were aligned using MAFFT (Katoh et al. 2017). Then, the maximum likelihood (ML) tree was constructed using IQ-TREE software (Kalyaanamoorthy et al. 2017; Hoang et al. 2018). The results indicated that *S. orbiculatus*, *L. japonica* and *Triosteum pinnatifidum* formed a monophyletic clade. Furthermore, there was a close relationship between *S. orbiculatus* and *L. japonica.*

**Figure 1.**
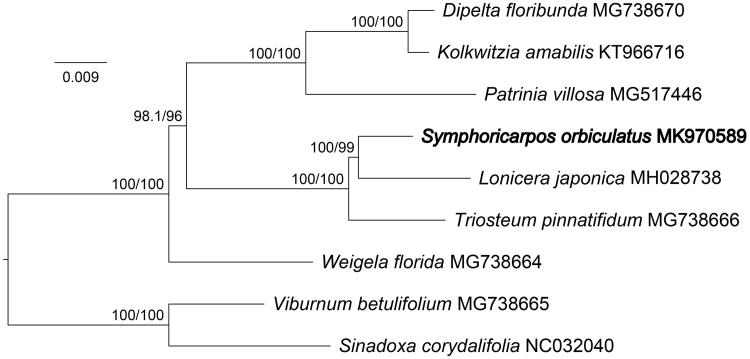
A maximum likelihood tree was constructed based on 7 Caprifoliaceae chloroplast genomes with 2 Adoxaceae genomes as outgroup species. The position of *Symphoricarpos orbiculatus* is shown in bold. Values above nodes are values of SH-aLRT branch test/bootstrap test.
